# Development and validation of non-invasive prediction models for migraine in Chinese adults

**DOI:** 10.1186/s10194-023-01675-1

**Published:** 2023-11-06

**Authors:** Shaojie Duan, Hui Xia, Tao Zheng, Guanglu Li, Zhiying Ren, Wenyan Ding, Ziyao Wang, Zunjing Liu

**Affiliations:** 1 Department of Geriatrics, Taizhou Central Hospital (Taizhou University Hospital), Taizhou, Zhejiang China; 2 The Second Clinical College, Guangzhou University of Chinese Medicine, Guangzhou, China; 3 Dongfang Hospital, Beijing University of Chinese Medicine, Beijing, China; 4 Graduate School of Beijing University of Chinese Medicine, Beijing, China; 5 Department of Neurology, China-Japan Friendship Hospital, Beijing, China; 6 Department of Neurology, Peking University People’s Hospital, Beijing, China

**Keywords:** Migraine, Prediction model, Receiver operating characteristic curve, Pittsburgh sleep quality index, Traditional Chinese medicine constitution

## Abstract

**Background:**

Migraine is a common disabling neurological disorder with severe physical and psychological damage, but there is a lack of convenient and effective non-invasive early prediction methods. This study aimed to develop a new series of non-invasive prediction models for migraine with external validation.

**Methods:**

A total of 188 and 94 subjects were included in the training and validation sets, respectively. A standardized professional questionnaire was used to collect the subjects' 9-item traditional Chinese medicine constitution (TCMC) scores, Pittsburgh Sleep Quality Index (PSQI) score, Zung's Self-rating Anxiety Scale and Self-rating Depression Scale scores. Logistic regression was used to analyze the risk predictors of migraine, and a series of prediction models for migraine were developed. Receiver operating characteristic (ROC) curve and calibration curve were used to assess the discrimination and calibration of the models. The predictive performance of the models were further validated using external datasets and subgroup analyses were conducted.

**Results:**

PSQI score and Qi-depression score were significantly and positively associated with the risk of migraine, with the area of the ROC curves (AUCs) predicting migraine of 0.83 (95% CI:0.77–0.89) and 0.76 (95% CI:0.68–0.84), respectively. Eight non-invasive predictive models for migraine containing one to eight variables were developed using logistic regression, with AUCs ranging from 0.83 (95% CI: 0.77–0.89) to 0.92 (95% CI: 0.89–0.96) for the training set and from 0.76 (95% CI: 0.66–0.85) to 0.83 (95% CI: 0.75–0.91) for the validation set. Subgroup analyses showed that the AUCs of the eight prediction models for predicting migraine in the training and validation sets of different gender and age subgroups ranged from 0.80 (95% CI: 0.63–0.97) to 0.95 (95% CI: 0.91–1.00) and 0.73 (95% CI: 0.64–0.84) to 0.93 (95% CI: 0.82–1.00), respectively.

**Conclusions:**

This study developed and validated a series of convenient and novel non-invasive prediction models for migraine, which have good predictive ability for migraine in Chinese adults of different genders and ages. It is of great significance for the early prevention, screening, and diagnosis of migraine.

**Supplementary Information:**

The online version contains supplementary material available at 10.1186/s10194-023-01675-1.

## Introduction

Migraine is the second most disabling neurological disorder and the third most prevalent medical condition in the world [1, 2], characterized by multiple episodes of moderate or severe headache and reversible neurological and systemic symptoms [3–5]. The prevalence of migraine varies by gender, with annual incidence rates of 6% and 18% for men and women, respectively, and lifetime rates of 13% and 33%, respectively [3]. In terms of age, the prevalence is similar for boys and girls before puberty, but in females it rises significantly after puberty, peaking between 35 and 39 years of age [4]. Migraine not only reduces a patient's health-related quality of life and leads to migraine-related disability, but also increases the risk of cardiovascular and cerebrovascular diseases, psychiatric disorders such as anxiety and depression, and has a significant impact on daily activities and direct medical costs [6–10]. Therefore, strengthening early prevention, diagnosis and timely and effective intervention of migraine is of great significance to patients, families and even society.

Despite the rapid development of international understanding of the pathophysiology of migraine and evidence-based guidelines designed to inform clinical decision-making in migraine, the prevention and treatment of migraine remains suboptimal, particularly in the early prevention and diagnosis of migraine [5, 11]. Because in reality, few people seek medical attention for mild headaches (e.g., stress- and tension-related headaches), which can lead to underdiagnosis of early migraine [12]. However, by the time patients arrive at the hospital seeking headache treatment, they have usually progressed to more severe migraines or chronic migraines of longer duration, which can lead to more severe migraine related burdens [12–14]. In recent years, many researchers have conducted preliminary explorations of diagnostic biomarkers for migraine, including those based on genetics, provocation modeling, biochemistry, and neuroimaging [15, 16], as well as a number of migraine-specific biomarkers using a "omics" approach [17]. However, most of the biomarkers in previous studies have limitations due to their invasiveness or high acquisition costs, which are not conducive to early diagnosis and population screening for migraine. Therefore, there is an urgent need for more convenient, efficient, safe, and noninvasive methods for early prediction and diagnosis of migraine.

Migraine is known to be associated with a variety of sleep disorders and psychiatric disorders, such as anxiety and depression, and the bidirectional relationship between migraine and these disorders is of increasing interest to researchers [7, 18–22]. Our previous studies showed that poor sleep quality, anxiety, and depression were significantly and positively associated with increased risk of migraine and migraine burdens, and that Pittsburgh Sleep Quality Index (PSQI) score, Zung's Self-rating Anxiety Scale (SAS) and Self-rating Depression Scale (SDS) scores had good predictive value for migraine and could be used as potential predictors for migraine [23, 24].

Traditional Chinese Medicine Composition (TCMC) has a complete functional state classification system, which is widely used in health care, subhealth prevention, quality of life evaluation, disease diagnosis, treatment and prevention [25–30]. A previous study analyzed 1,639 clinical studies on TCMC-disease correlations published over the past 10 years, including 19 disease categories and 333 different disorders, and showed that eight biased TCMCs were strongly associated with specific diseases and could be used to guide personalized prevention and treatment [26]. Another study found that Yang-deficiency constitution was closely related to metabolic syndrome and may be a potential predictor of metabolic syndrome [25]. In addition, a previous study found that TCMC was strongly associated with the risk of depression in women [31]. Based on the above findings and considering the close relationship between migraine and depression, we initially hypothesized that TCMC may be associated with the risk of migraine. As shown in a survey of TCMC characteristics in migraine patients in Hong Kong, Qi-depression and Blood-stasis constitutions were risk factors for migraine [32]. However, the predictive value of TCMC scores for migraine are unclear. In addition, it remains to be studied and explored whether the combination of TCMC scores with PSQI, SAS, and SDS scores can improve the predictive ability of migraine.

Therefore, this study will systematically explore the relationship between TCMC scores and the risk of migraine, evaluate the predictive value of different TCMC scores for migraine, and then screen out appropriate TCMC predictors and combine them with PSQI, SAS and SDS scores to establish novel non-invasive prediction models for migraine, and externally validate the prediction models, thereby providing new ideas and methods for early prevention and diagnosis of migraine.

## Methods and materials

### Study design and participants recruitment

This study utilized a case–control study method to develop and validate a new non-invasive prediction model for migraine in Chinese adults. The research design and paper writing referred to the TRIPOD reporting guidelines [33]. This study was approved by the Ethics Committee of Beijing University of Chinese Medicine (Project No.2022BZYLL0903). All subjects volunteered to participate in this study and signed an informed consent form.

**Fig. 1:**
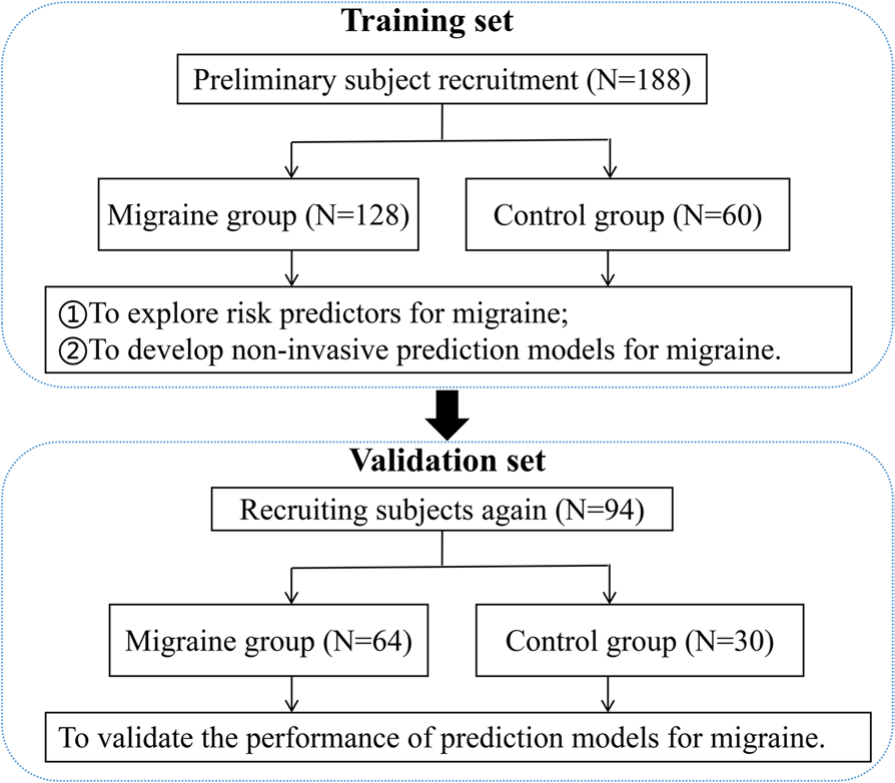
Research flowchart of this study

First, 128 migraine patients who met the research criteria and 60 sex- and age-matched healthy control subjects attending the headache clinic of the Department of Neurology at China-Japan Friendship Hospital between April 2021 and September 2022 were recruited continuously as a training set to establish the non-invasive prediction models for migraine. Then, from September 2022 to June 2023, 64 migraine patients who met the research criteria and 30 healthy control subjects were recruited continuously as the external validation set to further validate the predictive performance of the prediction models. The research flow chart of the subjects was shown in Fig. 1.

### Outcome and diagnosis criteria

The main outcome of this study was the presence or absence of migraines. All subjects underwent professional migraine diagnostic evaluation and completed a standardized questionnaire with interviews conducted by certified neurologists and headache specialists without knowing other independent variables and predictors influencing the participants. The diagnostic criteria for migraine were referenced to the International Classification of Headache Disorders (ICHD-3), third edition [34]. The migraine subjects included in this study included two types: migraine with aura and migraine without aura.

### Inclusion and exclusion criteria

Inclusion criteria of migraine subjects: 1) Fulfilled the diagnostic criteria for migraine (including migraine without aura and migraine with aura). 2) Aged between 18 and 65 years old, regardless of gender. 3) Had at least one migraine attack in the past month. 4) Those who volunteered to participate in this study and signed the informed consent form.

Exclusion criteria: 1) People under the age of 18 or over the age of 65, or pregnant or breastfeeding women. 2) People with serious primary diseases, such as heart, liver, kidney, blood system, mental diseases or malignant tumors. 3) People with infectious diseases, such as tuberculosis and AIDS. 4) Secondary headache caused by brain or other organic lesions. 5) Specific types of migraine, such as hemiplegic migraine, ophthalmoplegic migraine, and other migraines. 6) Subjects with missing or clearly incorrect important data.

In addition, all healthy control subjects were required to have no family history of migraine and no current or prior diagnosis of primary or secondary migraine.

### Measurement of main research indicators

Pittsburgh Sleep Quality Index (PSQI): The PSQI is a common self-assessment questionnaire to assess sleep quality [35]. It consists of 19 items across 7 components, and PSQI scores range from 0 to 21, with higher scores indicating poorer sleep quality [36]. Poor sleep quality is defined when the PSQI score is greater than 5, with a diagnostic sensitivity of 98.7 and specificity of 84.4 [37].

Zung's Self-rating Anxiety Scale (SAS) and Self-rating Depression Scale (SDS): The SAS and SDS are commonly used questionnaires for evaluating anxiety and depression, and their reliability and validity have been validated in Chinese populations [38–40]. Both scales include 20 items, with the original scale scores ranging from 20–80 and the transformed index scores ranging from 25–100, with higher scores indicating higher levels of anxiety and depression [41].

Traditional Chinese medicine constitution (TCMC): The TCMC includes nine subtypes, including Gentleness type, Qi-deficiency type, Yang-deficiency type, Yin-deficiency type, Phlegm-Wetness type, Wet-heat type, Blood-stasis type, Qi-depression type, and Special diathesis type, which were measured using Nine-Constitution Scale with an internal consistency of 0.72 to 0.82 and a retest reliability of 0.77 to 0.90 [31, 42]. The scale for each subtype of TCMC of 6 to 8 items, each with a score ranging from 0 to 4 [26, 27]. The raw scores were then converted to a score of 0 to 100, which was the TCMC score for that subtype.

### Other measurement

Demographic and baseline characteristics such as gender, age, height, weight, smoking history, drinking history, weekly exercise time, and subjective pressure score were collected through standardized questionnaire interviews and quality control. Body mass index (BMI) was calculated as weight in kilograms divided by the square of height in meters. Smoking history and drinking history were defined as current or previous smoking and drinking behavior. Weekly exercise time was defined as total weekly exercise time (hours). Subjective stress was assessed by a visual analog pressure scale ranging from 0 to 10.

### Statistical analysis

This study was a preliminary exploratory study, where the sample size of the training and validation sets was set in a 2:1 ratio and met the minimum sample size required for statistical methods to develop predictive models. Samples with missing important data have been excluded. Statistical analyses and figure plotting were performed using SPSS, version 25.0, the Python package, version sklearn 0.22.1, and the R package, version logreg6.2.0. All statistical tests were two-tailed and were considered significant for P less than 0.05 (*P* values < 0.05).

First, baseline characteristics of the migraine and control groups in the training set were compared. The chi-square test was used to compare categorical data between groups, expressed as number of cases and percentage [n (%)]. The independent samples t-test was used to compare quantitative data that were normally distributed between groups, expressed as mean ± standard deviation (SD). The Mann–Whitney U-test was used to compare quantitative data that were not normally distributed between groups, expressed as median [interquartile range (IQR)]. Then, logistic regression analyses were performed to assess the effects of PSQI scores, SAS scores, SDS scores, and the nine TCMC scores on the risk of migraine under different adjustment conditions, to screen the risk predictors of migraine, and to establish the optimal prediction models for migraine that included different numbers of indicators. In addition, receiver operating characteristic curves (ROCs) were plotted to assess the discrimination of predition models, and the area under the ROC curves (AUCs) of predition models in the total sample and in subgroups of different genders and ages were compared by the Delong test. Calibration curves and the Hosmer–Lemeshow test were used to assess the calibration of the prediction models. Finally, we validated the ability of each prediction model in an external validation set.

## Results

### Baseline characteristics of participants

The baseline characteristics of training and validation sets were presented in Table 1. From the training set, it can be seen that there were no significant difference in gender, age, BMI, smoking history, drinking history, weekly exercise time, pressure score and the Yin-deficiency score, Phlegm-wetness score, Wet-heat score, and Special diathesis score levels between the migraine group and the control group (all *P* values > 0.05). The PSQI score, SAS score, SDS score, Qi-deficiency score, Yang-deficiency score, Blood-stasis score, and Qi-depression score levels of the migraine group were significantly higher than those of the control group, while the Gentleness score level was significantly lower than that of the control group (all *P* values < 0.05). The same results were found for the validation set. There were no statistically significant differences between the variables in the validation set and the training set (all *P* values > 0.05), indicating that the validation set was well represented and comparable.

**Table 1:** Comparison of baseline data between training and validation sets

Variable	Training set			Validation set			*P*1	*P*2	*P*3
	Total(*N* = 188)	Control(*N* = 60)	Migraine(*N* = 128)	Total(*N* = 94)	Control(*N* = 30)	Migraine(*N* = 64)			
Gender, male, n(%)	27(14.36)	10(16.67)	17(13.28)	17(18.09)	5(16.67)	12(18.75)	0.537	0.807	0.417
Gender, female, n(%)	161(85.64)	50(83.33)	111(86.72)	77(81.91)	25(83.33)	52(81.25)			
Age, years, median[IQR]	35.00[31.00,41.00]	34.00[29.00,37.00]	36.00[31.00,42.00]	37.00[32.00,43.00]	37.00[33.00,45.00]	38.00[32.00,42.00]	0.164	0.742	0.096
BMI, kg/m^2^, median[IQR]	21.88[19.83,22.99]	22.01[19.88,23.56]	21.23[19.81,22.76]	21.51[19.59,23.95]	21.27[19.24,22.83]	21.83[20.07,24.22]	0.392	0.316	0.366
Smoking history, n(%)	18(9.57)	4(6.67)	14(10.94)	10(10.64)	1(3.33)	9(14.06)	0.354	0.116	0.778
Drinking history, n(%)	39(20.74)	11(18.33)	28(21.88)	19(20.21)	6(20.00)	13(20.31)	0.577	0.972	0.917
Weekly exercise time, hours, median[IQR]	0.00[0.00,1.00]	0.00[0.00,0.67]	0.00[0.00,1.50]	0.00[0.00,1.33]	0.00[0.00,2.00]	0.00[0.00,1.17]	0.077	0.680	0.817
Pressure score, mean(± SD)	5.10 ± 2.46	5.49 ± 2.37	4.93 ± 2.49	5.26 ± 2.37	4.93 ± 2.46	5.40 ± 2.32	0.154	0.387	0.624
PSQI score, median[IQR]	6.00[4.00,8.00]	4.00[3.00,5.00]	7.00[5.00,9.00]	5.00[4.00,8.00]	4.00[3.00,5.00]	7.00[4.00,9.00]	< 0.001	< 0.001	0.199
SAS score, median[IQR]	38.75[33.75,46.25]	33.75[31.25,37.50]	42.50[36.25,48.75]	38.75[32.50,46.25]	33.75[30.00,40.00]	42.50[35.00,47.50]	< 0.001	< 0.001	0.799
SDS score, median[IQR]	38.75[33.75,48.75]	37.50[33.75,41.25]	40.00[33.75,51.25]	38.75[32.50,50.00]	33.75[28.75,42.50]	41.25[35.00,52.50]	0.017	< 0.001	0.888
TCMC score									
Gentleness score, mean(± SD)	62.42 ± 18.29	70.73 ± 15.88	58.52 ± 18.05	62.27 ± 19.27	73.86 ± 16.99	56.84 ± 17.81	< 0.001	< 0.001	0.942
Qi-deficiency score, median[IQR]	25.00[15.63,40.63]	18.75[9.38,21.88]	31.25[18.75,46.88]	25.00[15.63,37.50]	18.75[3.13,28.13]	28.13[21.88,40.63]	< 0.001	< 0.001	0.642
Yang-deficiency score, median[IQR]	28.13[10.71,46.43]	15.63[3.13,32.14]	28.57[14.29,50.00]	25.00[10.71,42.86]	15.63[7.14,28.13]	32.14[14.29,53.57]	0.004	0.002	0.951
Yin-deficiency score, median[IQR]	21.88[9.38,34.38]	18.75[9.38,31.25]	21.88[12.50,34.38]	25.00[6.25,37.50]	15.63[6.25,31.25]	28.13[9.38,40.63]	0.128	0.066	0.985
Phlegm-wetness score, median[IQR]	25.00[12.50,40.63]	25.00[12.50,43.75]	25.00[15.63,40.63]	25.00[12.50,40.63]	25.00[12.50,34.38]	25.00[12.50,43.75]	0.598	0.503	0.968
Wet-heat score, median[IQR]	25.00[12.50,45.83]	25.00[12.50,45.83]	29.17[16.67,41.67]	25.00[12.50,41.67]	29.17[16.67,45.83]	25.00[12.50,41.67]	0.635	0.542	0.949
Blood-stasis score, median[IQR]	25.00[10.71,35.71]	17.86[7.14,25.00]	28.57[17.86,39.29]	21.43[10.71,32.14]	14.29[10.71,25.00]	25.00[10.71,35.71]	< 0.001	0.026	0.308
Qi-depression score, median[IQR]	21.43[7.14,42.86]	7.14[3.57,21.43]	28.57[10.71,42.86]	21.43[7.14,35.71]	7.14[3.57,17.86]	32.14[14.29,39.29]	< 0.001	< 0.001	0.915
Special diathesis score, median[IQR]	17.86[7.14,32.14]	17.86[14.29,32.14]	17.86[7.14,32.14]	17.86[7.14,32.14]	14.29[7.14,21.43]	17.86[7.14,32.14]	0.300	0.240	0.511

*P*_1_ indicated the comparison between the migraine group and the control group in the training set; *P*_2_ indicated the comparison between the migraine group and the control group in the validation set; *P*_3_ indicated the comparison of the total samples between the training and validation sets.Chi-squared test, t test and Mann–whitney U test were used for statistical analysis, which were expressed as n (%), mean (± SD), and medium [IQR], respectively. *SD* Standard deviation, *IQR* Interquartile range, *BMI* Body mass index, *PSQI* Pittsburgh sleep quality index, *SAS* Self-rating anxiety scale, *SDS* Self-rating depression scale, *TCMC* Traditional Chinese medicine constitution

### Risk predictors for developing migraine

Logistic regression analyses were conducted to explore the effects of PSQI score, SAS score, SDS score and nine TCMC scores on the risk of developing migraine. It can be seen from Table 2 that the PSQI score, SAS score, SDS score, Gentleness score, Qi-deficiency score, Blood-stasis score, and Qi-depression score were significantly correlated with the risk of migraine (all *P* values < 0.05). After adjusting for gender, age, smoking history, drinking history, BMI, weekly exercise time, and pressure score, the odds ratios (ORs) for a 1-standard deviation (SD) increase in PSQI score, SAS score, SDS score, Gentleness score, Qi-deficiency score, Blood-stasis score, and Qi-depression score were still 1.748 (95% CI: 1.436–2.128), 1.139 (95% CI: 1.081–1.200), 1.042 (95% CI: 1.007–1.079), 0.959 (95% CI: 0.938–0.979), 1.053 (95% CI: 1.027–1.078), 1.034 (95% CI: 1.011–1.058) and 1.047 (95% CI: 1.025–1.069), respectively.

**Table 2:** Results of logistic regression analysis and AUCs on the risk of migraine by various variables

Variable	Unadjusted		Adjusted^*^		AUC(95%CI)
	OR (95% CI)	*P* value	OR (95% CI)	*P* value	
PSQI score	1.772(1.464–2.145)	< 0.001	1.748(1.436–2.128)	< 0.001	0.83(0.77–0.89)
SAS score	1.130(1.077–1.186)	< 0.001	1.139(1.081–1.200)	< 0.001	0.75(0.68–0.83)
SDS score	1.042(1.009–1.076)	0.013	1.042(1.007–1.079)	0.018	0.61(0.52–0.69)
Gentleness score	0.960(0.941–0.979)	< 0.001	0.959(0.938–0.979)	< 0.001	0.70(0.62–0.77)
Qi-deficiency score	1.053(1.029–1.077)	< 0.001	1.053(1.027–1.078)	< 0.001	0.70(0.62–0.78)
Yang-deficiency score	1.017(1.003–1.031)	0.016	1.012(0.998–1.027)	0.101	0.63(0.54–0.72)
Yin-deficiency score	1.019(0.999–1.038)	0.059	1.017(0.996–1.038)	0.116	0.57(0.48–0.66)
Phlegm-wetness score	0.995(0.980–1.011)	0.535	0.994(0.978–1.011)	0.485	0.52(0.44–0.62)
Wet-heat score	1.000(0.985–1.015)	0.953	0.999(0.984–1.015)	0.950	0.52(0.43–0.62)
Blood-stasis score	1.034(1.013–1.056)	0.001	1.034(1.011–1.058)	0.004	0.67(0.59–0.76)
Qi-depression score	1.046(1.026–1.067)	< 0.001	1.047(1.025–1.069)	< 0.001	0.76(0.68–0.84)
Special diathesis score	0.994(0.976–1.011)	0.474	0.993(0.974–1.012)	0.467	0.55(0.46–0.63)

^*^Adjusted for age, gender, smoking history, drinking history, BMI, weekly exercise time, pressure score. *PSQI* Pittsburgh sleep quality index, *SAS* Self-rating anxiety scale, *SDS* Self-rating depression scale, *AUC* Area under the receiver operating characteristic curve

In addition, Table 2 also illustrated the AUCs of these 12 variables for predicting migraine. The results showed that the top three indicators for ranking predictive ability were PSQI score, Qi-depression score, and SAS score, with all AUCs greater than 0.7, indicating good predictive ability for migraine.

### Establishment of non-invasive risk prediction models for developing migraine

First, PSQI score [AUC = 0.83 (95% CI: 0.77–0.89)], the best predictor of migraine among all predictors, was selected and included in Logistic regression to build a prediction model for migraine containing only one variable (Model 1). Then, PSQI score, SAS score, SDS score, Gentleness score, Qi-deficiency score, Blood-stasis score, and Qi-depression score, which were significantly associated with the risk of migraine, were further included in the progressive forward Logistic regression. The result showed that only PSQI score and Qi-depression score were still significantly and independently associated with the increased risk of migraine. Based on this, a Logistic regression prediction model (model 2) containing these two variables was constructed.

In addition, in order to further improve the prediction performance for migraine, we also tried to incorporate PSQI score, SAS score, SDS score and nine TCMC scores into the progressive forward Logistic regression in various combinations, and screened out the best prediction models containing 3 to 8 variables, respectively, model 3 to model 8. The specific Logistic regression equation and predictive performance results of each model were illustrated in Table 3. Meanwhile, it also provided the optimal cutoff values for each model, which can be used in clinical applications to predict whether there is migraine.

**Table 3:** Prediction performance results of various models in training set

Model ^*^	No. of variables	AUC(95%CI)	Sensitivity	Specificity	Youden index	Cut off value	*P* for H–L test ^#^
Model 1	1	0.83(0.77–0.89)	0.820	0.683	0.504	0.621	0.923
Model 2	2	0.86(0.80–0.91)	0.789	0.833	0.622	0.640	0.390
Model 3	3	0.87(0.82–0.92)^a^	0.781	0.833	0.615	0.653	0.688
Model 4	4	0.88(0.84–0.93)^ab^	0.867	0.767	0.634	0.569	0.289
Model 5	5	0.90(0.85–0.94)^ab^	0.813	0.850	0.663	0.698	0.873
Model 6	6	0.91(0.86–0.95)^abcd^	0.695	0.950	0.645	0.828	0.148
Model 7	7	0.91(0.87–0.95)^abcd^	0.766	0.900	0.666	0.762	0.632
Model 8	8	0.92(0.89–0.96)^abcd^	0.922	0.800	0.722	0.494	0.049

^*^Model 1: logit(p1) = -2.365 + 0.572 × "PSQI score"; Model 2: logit(p2) = -2.954 + 0.547 × "PSQI score" + 0.034 × "Qi-depressed score"; Model 3: logit(p3) = -2.606 + 0.599 × "PSQI score" + 0.046 × "Qi-depressed score"-0.039 × "Inherited special score"; Model 4: logit(p4) = -2.667 + 0.581 × "PSQI score" + 0.039 × "Qi-depressed score" + 0.034 × "Blood-stasis score"-0.05 × "Phlegm-dampness score"; Model 5: logit(p5) = -2.267 + 0.554 × "PSQI score" + 0.046 × "Qi-depressed score" + 0.041 × "Qi-deficient score"-0.04 × "Phlegm-dampness score"-0.043 × "Inherited special score"; Model 6: logit(p6) = -2.446 + 0.574 × "PSQI score" + 0.036 × "Qi-depressed score" + 0.039 × "Qi-deficient score" + 0.048 × "Blood-stasis score"-0.059 × "Phlegm-dampness score"-0.055 × "Inherited special score"; Model 7: logit(p7) = -0.84 + 0.612 × "PSQI score"-0.057 × "SDS score" + 0.046 × "Qi-depressed score" + 0.052 × "Qi-deficient score" + 0.051 × "Blood-stasis score"-0.063 × "Phlegm-dampness score"-0.057 × "Inherited special score"; Model 8: logit(p8) = -2.687 + 0.532 × "PSQI score" + 0.12 × "SAS score"-0.111 × "SDS score" + 0.043 × "Qi-depressed score" + 0.055 × "Qi-deficient score" + 0.051 × "Blood-stasis score"-0.068 × "Phlegm-dampness score"-0.058 × "Inherited special score"

^#^indicated the *P* value for the Hosmer–Lemeshow test for various model

^a^indicated that after Delong test, the AUC was significantly better than model 1

^b^indicated that after Delong test, the AUC was significantly better than model 2

^c^indicated that after Delong test, the AUC was significantly better than model 3

^d^indicated that after Delong test, the AUC was significantly better than model 4

*AUC* Area under the receiver operating characteristic curve, *H–L* Hosmer–Lemeshow

### Evaluation of discrimination and calibration of prediction models

**Fig. 2:**
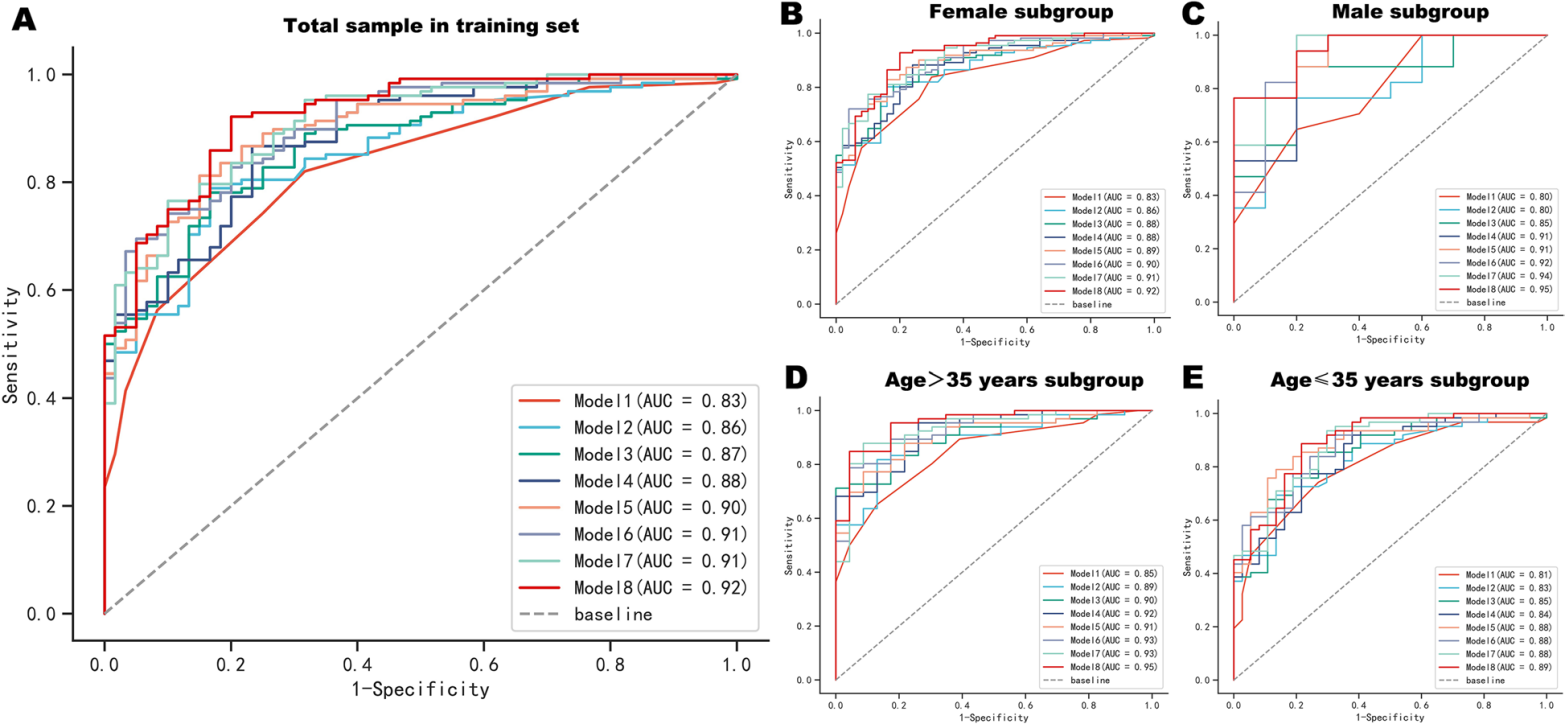
ROC curves of eight prediction models for the total sample (**A**) and different subgroups (**B-E**) in the training set. In the training set, the AUC for models 1 to 8 ranged from 0.83 (95% CI:0.77–0.89) to 0.92 (95% CI:0.89–0.96) in the total sample and 0.80 (95% CI:0.63–0.97) to 0.95 (95% CI:0.91–1.00) in the different sex and age subgroups. *AUC* Area under the ROC curve

First, in order to evaluate the discrimination of these prediction models, the ROC curves of eight prediction models were drawn (Fig. 2A), and the Delong test was conducted to compare the prediction ability of each model. The results showed that the AUCs of models 1 to 8 were 0.83 (95%CI: 0.77–0.89), 0.86 (95%CI: 0.80–0.91), 0.87 (95%CI: 0.82–0.92), 0.88 (95%CI: 0.84–0.93), 0.90 (95%CI: 0.85–0.94), 0.91 (95%CI: 0.86–0.95), 0.91 (95%CI: 0.87–0.95), and 0.92 (95%CI: 0.89–0.96), respectively. Compared to Model 1, the AUCs of Models 3 to 8 with 3 to 8 variables had significantly improved. Compared to Model 2, the AUCs of Models 4 to 8 with 4–8 variables had significantly improved. In addition, the AUCs of Model 6 to Model 8 were significantly higher than those of Model 1 to Model 4 (all *P* values < 0.05), and all AUCs were not less than 0.9. It was worth noting that Model 6 also had the best specificity (0.95) and Model 8 had the best sensitivity (0.92).

**Fig. 3:**
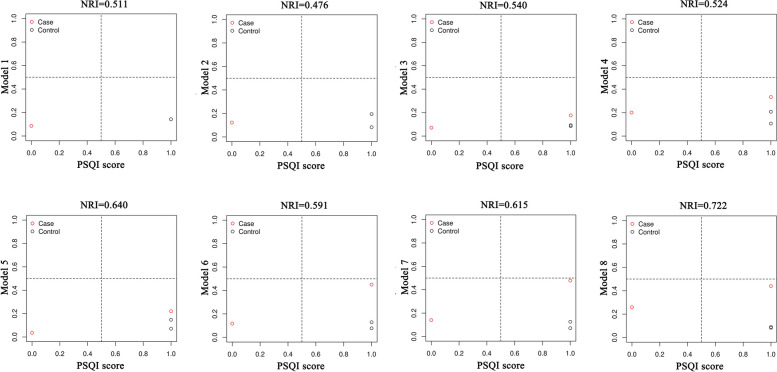
Plots of NRI analysis results between the eight models and the PSQI score predictor in the training set. Compared to the PSQI score, the correct reclassification rate from Model 1 to Model 8 increased by 51.1%, 47.6%, 54.0%, 52.4%, 64.0%, 59.1%, 61.5%, and 72.2%, respectively. *NRI* Net reclassification index, *PSQI* Pittsburgh sleep quality index

A net reclassification index (NRI) analysis was then performed to compare the diagnostic accuracy of the eight models with the single best predictor, PSQI score. The results, as shown in Fig. 3 and Supplementary Tables 1–8, showed that the percentage of correct reclassification from model 1 to model 8 was improved by 51.1%, 47.6%, 54.0%, 52.4%, 64.0%, 59.1%, 61.5%, and 72.2%, respectively, in comparison with PSQI score, which indicated that the prediction accuracies of the eight models established in the present study for migraine were all greatly improved and significantly better than the PSQI score.

**Fig. 4:**
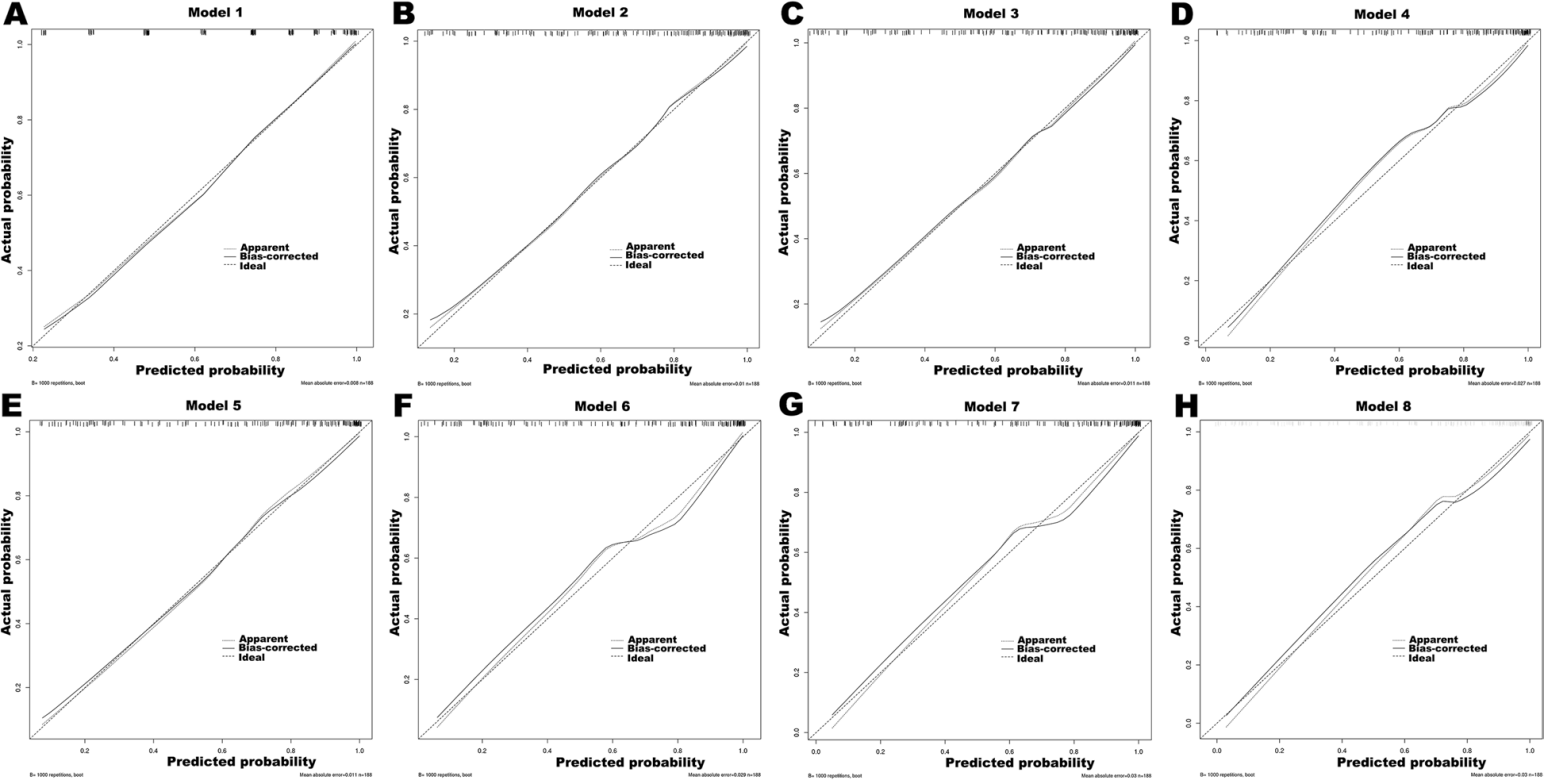
Calibration curves of eight prediction models for migraine in the training set. The Hosmer–Lemeshow tests showed that models 1 to 7 had excellent calibration (*P* for H–L test > 0.05), but the calibration of model 8 was not very good (*P* for H–L test = 0.049 < 0.05). *H–L* Hosmer–Lemeshow

In addition, in order to evaluate the calibration of these prediction models, calibration curves were drawn (Fig. 4) and the Hosmer–Lemeshow tests were performed on each model. The results showed that models 1 to 7 had excellent calibration (*P* for H–L test > 0.05), but the calibration of model 8 was not very good (*P* for H–L test = 0.049 < 0.05).

### Collinearity diagnosis of prediction model variables

**Fig. 5:**
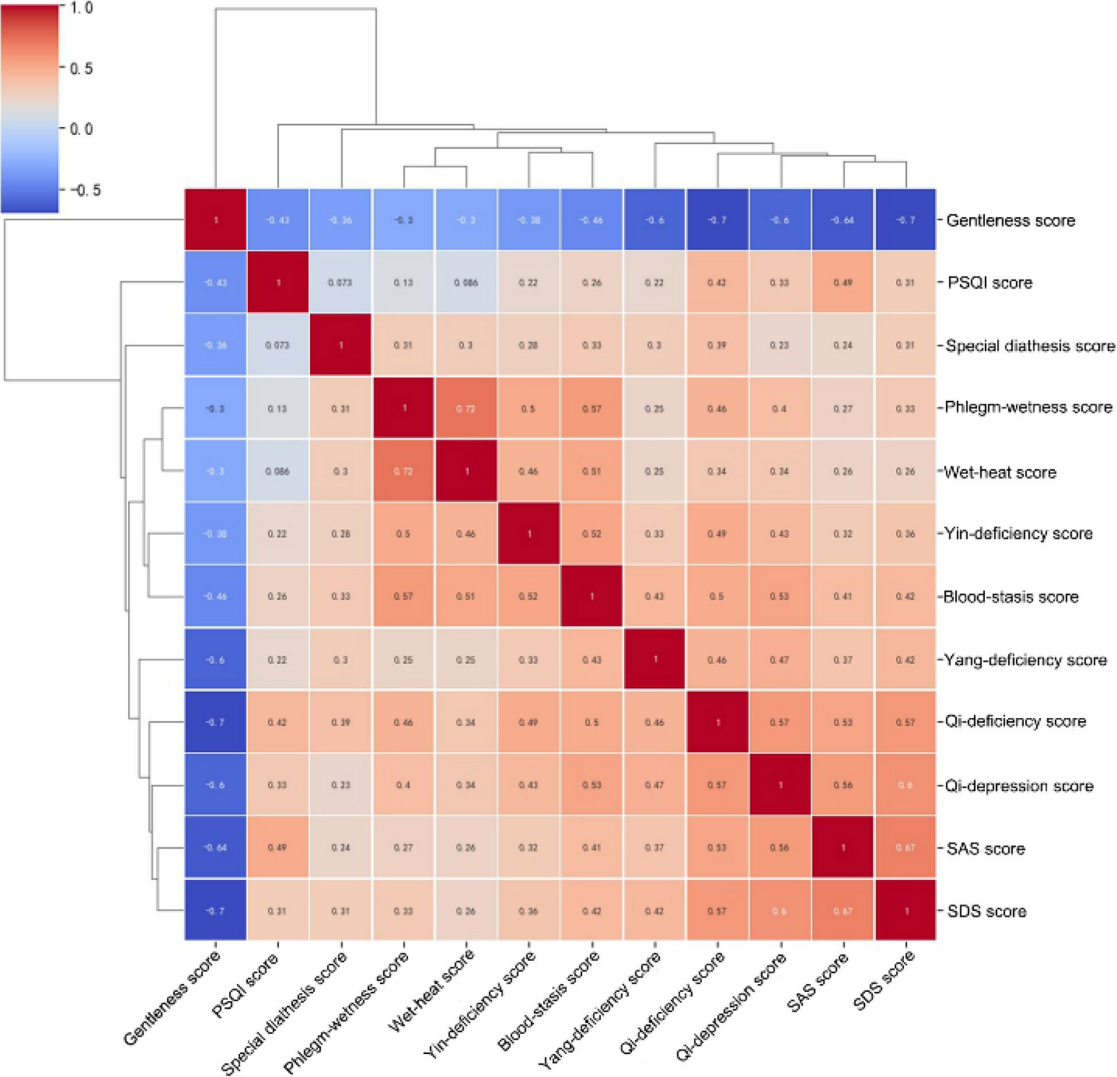
Correlation cluster graph of each variable in the training set

Considering that the variables included in some models were not significant in the Logistic regression analysis results in Table 2, or the positive and negative values of the variables have changed, there may be a problem of variable collinearity. Therefore, we specifically drew a correlation cluster graph (Fig. 5) for each variable and conducted collinearity diagnosis on the variables included in each model. The results showed in Table 4 that the variance inflation factor (VIF) of all variables in the eight models were less than 5, indicating that there were no multicollinearity problem and these prediction models were well constructed.

**Table 4:** Collinearity diagnosis of various model variables

Variable	VIF^*^							
	Model1	Model 2	Model 3	Model4	Model5	Model6	Model7	Model8
PSQI score	1	1.09	1.09	1.1	1.2	1.2	1.22	1.39
SAS score	——	——	——	——	——	——	——	2.53
SDS score	——	——	——	——	——	——	1.9	2.48
Qi-deficient score	——	——	——	——	1.85	1.86	2	2.02
Phlegm-dampness score	——	——	——	1.46	1.34	1.55	1.55	1.56
Blood-stasis score	——	——	——	1.67	——	1.73	1.73	1.73
Qi-depressed score	——	1.09	1.17	1.48	1.56	1.7	2.01	2.07
Inherited special score	——	——	1.08	——	1.21	1.23	1.25	1.25

^*^VIF was used to evaluate the multicollinearity of model variables. When VIF > 5, it indicates that there was Multicollinearity, and when VIF < 5, there was no Multicollinearity. *VIF* Variance inflation factor, *PSQI* Pittsburgh sleep quality index, *SAS* Self-rating anxiety scale, *SDS* Self-rating depression scale

### Subgroup analysis of prediction models

Considering the epidemiological differences of migraine in terms of gender and age, we further conducted subgroup analysis according to gender and median age (35 years old), then the ROC curves of eight prediction models in different subgroups were drawn (Fig. 2B**-**E) and the Delong test was conducted to compare the prediction ability of each model in each subgroup. The results were shown in Table 5. The AUCs of eight models in each subgroup range from 0.80 (95% CI: 0.63–0.97) to 0.95 (95% CI: 0.91–1.00), indicating that all eight models have good predictive value for migraine in different gender and age subgroups. And Delong test showed that Model 6, Model 7, and Model 8 exhibited relatively good AUCs in any subgroup, consistent with the predicted results in the total sample of training set.

**Table 5:** Subgroup analysis of prediction performance of various models in training set

Subgroup	Model	N	AUC(95%CI)	Sensitivity	Specificity	Youden index	Cut off value
Gender							
Male	Model1	27	0.80(0.63–0.97)	0.647	0.800	0.447	0.744
	Model2	27	0.80(0.63–0.97)	0.765	0.800	0.565	0.507
	Model3	27	0.85(0.70–0.99)	0.882	0.800	0.682	0.512
	Model4	27	0.91(0.78–1.00)	1	0.800	0.800	0.365
	Model5	27	0.91(0.78–1.00)	1	0.700	0.700	0.412
	Model6	27	0.92(0.80–1.00)^a^	1	0.800	0.800	0.333
	Model7	27	0.94(0.84–1.00)^abc^	1	0.800	0.800	0.377
	Model8	27	0.95(0.87–1.00)^abc^	0.765	1	0.765	0.793
Female	Model1	161	0.83(0.77–0.90)	0.838	0.700	0.538	0.621
	Model2	161	0.86(0.81–0.92)	0.802	0.840	0.642	0.640
	Model3	161	0.88(0.83–0.93)^a^	0.811	0.820	0.631	0.653
	Model4	161	0.88(0.83–0.93)^a^	0.883	0.760	0.643	0.584
	Model5	161	0.89(0.84–0.94)^a^	0.829	0.840	0.669	0.698
	Model6	161	0.90(0.86–0.95)^ab^	0.721	0.960	0.681	0.830
	Model7	161	0.91(0.86–0.95)^ab^	0.775	0.900	0.675	0.762
	Model8	161	0.92(0.88–0.96)^abcd^	0.928	0.800	0.728	0.494
Age							
> 35 years	Model1	89	0.85(0.76–0.93)	0.652	0.870	0.521	0.837
	Model2	89	0.89(0.82–0.96)	0.818	0.870	0.688	0.731
	Model3	89	0.90(0.84–0.96)	0.712	1	0.712	0.852
	Model4	89	0.92(0.86–0.98)^a^	0.955	0.739	0.694	0.569
	Model5	89	0.91(0.84–0.97)	0.773	0.913	0.686	0.808
	Model6	89	0.93(0.87–0.99)^a^	0.788	0.957	0.744	0.841
	Model7	89	0.93(0.87–0.99)^a^	0.879	0.913	0.792	0.780
	Model8	89	0.95(0.91–1.00)^a^	0.848	0.957	0.805	0.815
≤ 35 years	Model1	99	0.81(0.72–0.90)	0.742	0.730	0.472	0.621
	Model2	99	0.83(0.75–0.91)	0.694	0.865	0.558	0.640
	Model3	99	0.85(0.77–0.93)	0.855	0.730	0.585	0.512
	Model4	99	0.84(0.77–0.92)	0.774	0.784	0.558	0.571
	Model5	99	0.88(0.82–0.95)^ab^	0.790	0.865	0.655	0.622
	Model6	99	0.88(0.81–0.95)^ab^	0.839	0.757	0.595	0.541
	Model7	99	0.88(0.82–0.95)^abd^	0.935	0.703	0.638	0.377
	Model8	99	0.89(0.83–0.96)^abcd^	0.887	0.784	0.671	0.494

^a^indicated that after Delong test, the AUC was significantly better than model 1

^b^indicated that after Delong test, the AUC was significantly better than model 2

^c^indicated that after Delong test, the AUC was significantly better than model 3

^d^indicated that after Delong test, the AUC was significantly better than model 4

*AUC* Area under the receiver operating characteristic curve

### External validation of prediction models

**Fig. 6:**
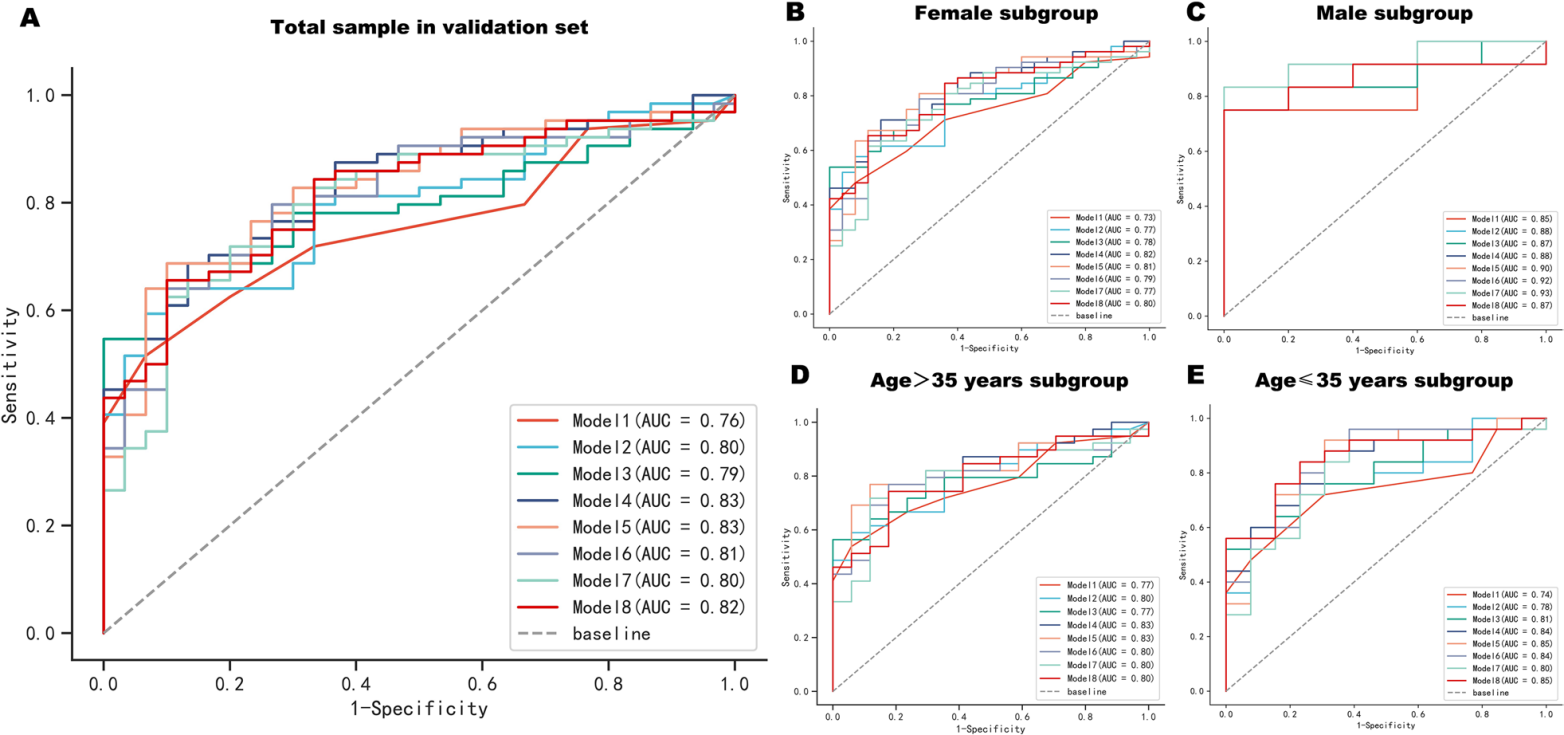
ROC curves of eight prediction models for the total sample (**A**) and different subgroups (**B-E**) of migraine in the validation set. In the validation set, the AUC for models 1 to 8 ranged from 0.76 (95% CI: 0.66–0.85) to 0.83 (95% CI: 0.75–0.91) in the total sample and 0.73 (95% CI: 0.64–0.84) to 0.93 (95% CI: 0.82–1.00) in the different sex and age subgroups. *AUC* Area under the ROC curve

In order to evaluate the applicability of the prediction models, 94 subjects with no statistically differences from the baseline data of the training set samples were recruited as the external validation set. The ROC curves of eight prediction models for migraine in the validation set were drawn to evaluate their prediction performance. As shown in Fig. 6A, the AUCs of models 1 to 8 were 0.76 (95% CI: 0.66–0.85), 0.80 (95% CI: 0.71–0.88), 0.79 (95% CI: 0.70–0.88), 0.83 (95% CI: 0.75–0.91), 0.83 (95% CI: 0.75–0.91), 0.81 (95% CI: 0.73–0.90), 0.80 (95% CI: 0.71–0.89), and 0.82 (95% CI: 0.74–0.90), respectively. In addition, Fig. 6B**-**E also showed the ROC curves of eight prediction models for different gender and age subgroups in the validation set. The results showed that the AUCs of these eight prediction models ranged from 0.73 (95% CI: 0.64–0.84) to 0.93 (95% CI: 0.82–1.00) in different subgroups. Therefore, the eight non-invasive prediction models established in this study showed excellent predictive ability in the overall population and subgroups of different genders and ages in the external validation set.

**Fig. 7:**
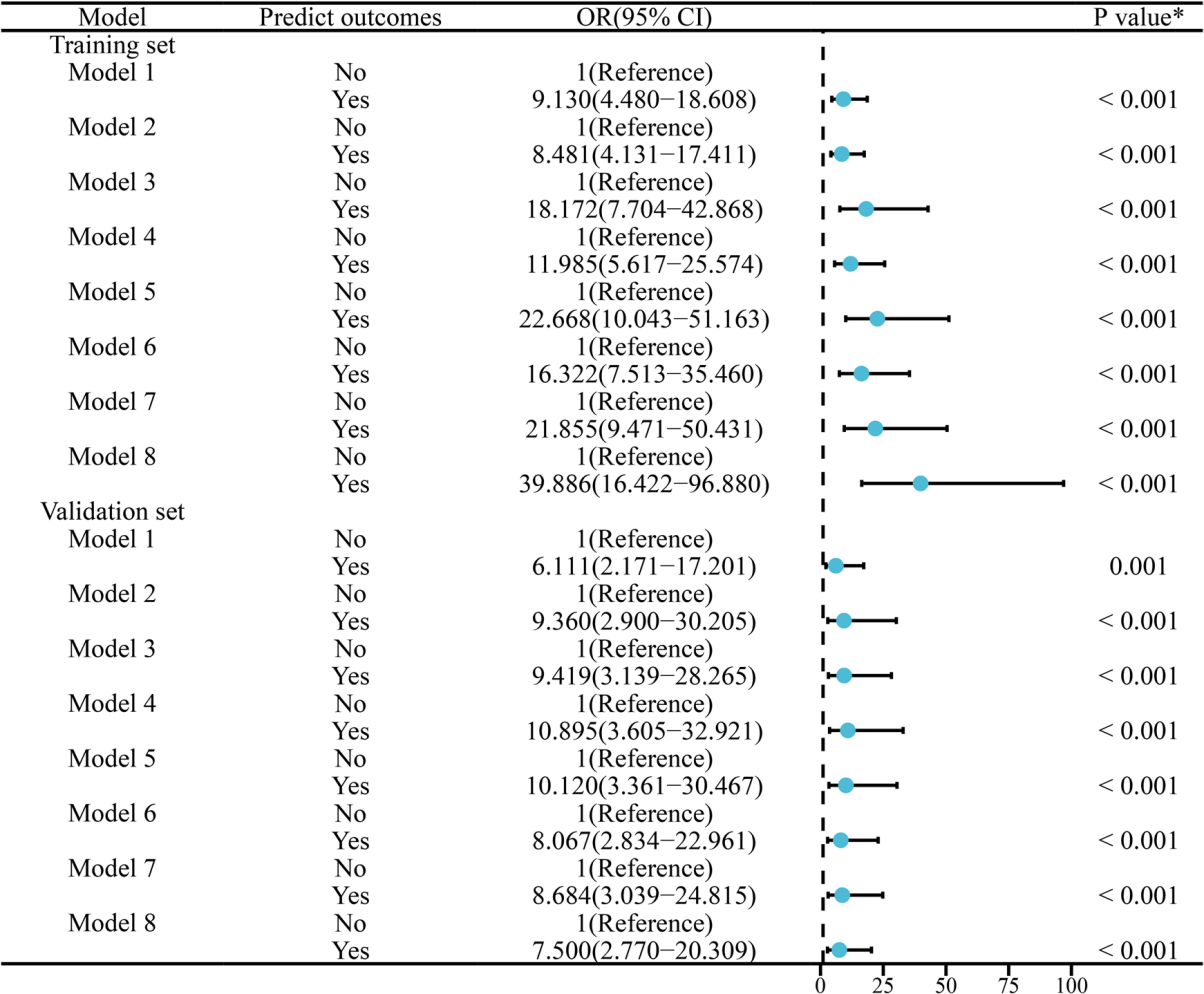
Effects of predict outcomes of eight models on the risk of migraine in training and validation sets. ^*^Adjusted for age, gender, smoking history, drinking history, BMI, weekly exercise time, pressure score

In addition, we also predicted whether migraine occurred or not based on the best cutoff values of the eight models in the training set, and further analyzed the association between the predict outcomes and the actual occurrence of migraine in the training and validation sets by multifactor logistic regression, respectively. The results showed that after adjusting for confounders such as age, gender, smoking history, drinking history, BMI, weekly exercise time, pressure score, the predict outcomes of eight models in both the training and validation sets were significantly and independently positively associated with the actual occurrence of migraine. As shown in Fig. 7, the ORs in the training set ranged from 8.481 (95% CI: 4.131–17.411) to 39.886 (95% CI: 16.422–96.880), and those in the validation set ranged from 6.111 (95% CI: 2.171–17.201) to 10.895 (95% CI: 3.605–32.921), which further confirmed the excellent predictive performance of the eight prediction models for migraine.

## Discussion

This study first systematically explored the relationship between PSQI score, SAS score, SDS score, and nine TCMC scores with the risk of migraine, and evaluated their predictive ability for migraine. The results not only confirmed the predictive value of PSQI score, SAS score, SDS score for migraine, but also found that the Qi-deficiency score, Blood-stasis score, and Qi-depression score were significant associated with the risk of migraine, and had a certain predictive ability for migraine. Then, this study mainly developed eight non-invasive prediction models for migraine, evaluated the discrimination and calibration of each prediction model, conducted subgroup analysis based on gender and age stratification, and validated the excellent predictive performance of each prediction model on the overall sample and different subgroup subjects using external datasets, providing new ideas and reliable methods for early prevention and diagnosis of migraine, which had important clinical application value.

To analyze the risk factors of developing migraine, seven predictors were preliminary screened out by multiple Logistic regression analysis, and then they were further included in the progressive forward logistic regression analysis. Finally, PSQI score and Qi depression score were demonstrated as significant independent risk factors of migraine. Two of our previous studies found that the PSQI score, SAS score, and SDS score were all significantly and positively associated with the risk of migraine, with the PSQI score having the relatively best predictive power for migraine [23, 24]. The results of this study showed that the AUCs of PSQI score, SAS score, and SDS score for migraine were 0.83 (95% CI: 0.77–0.89), 0.75 (95% CI: 0.68–0.83), and 0.61 (95% CI: 0.52–0.69), respectively, which were similar to our previous studies and further demonstrated that the PSQI score had the relatively best predictive ability for migraine. These results also further confirmed the close relationship between anxiety, depression and poor sleep quality and migraine.

In recent years, TCMC has shown significant value in many studies of psychosomatic and cardiovascular diseases. For example, a systematic evaluation study of TCMC as a predictor of depression showed that Qi-deficiency constitution and Qi-depression constitution could be used as a predictor of depression [43]. A cross-sectional study during the COVID-19 pandemic showed that Qi-deficiency constitution and Qi-depression constitution were associated with depression and Qi-depression constitution was associated with anxiety in patients with systemic sclerosis [44]. A study of Malaysian university students also showed that Qi-depression constitution was an important risk factor for depression among university students [45]. Another study has shown that Qi-deficiency was significantly associated with emotional, pain, and fatigue in SLE patients [46]. Moreover, Qi-deficiency constitution, Qi-depression constitution, and Blood-stasis constitution were all significantly associated with insomnia [47]. In addition, other studies have shown that Blood-stasis constitution was a risk factor for cognitive dysfunction [48], was strongly associated with the development of peripheral arterial disease in patients with type 2 diabetes mellitus [49], and may also be used as an early predictive diagnostic indicator for the development of coronary artery disease in patients with chest pain [50]. Considering that migraine, as a neurological disorder that is closely related to psychosomatic disorders, we hypothesized that TCM might also be closely related to migraine. The results of this study showed that the Qi-deficiency score, Blood-stasis score, and Qi-depression score were significantly and positively associated with the risk of migraine, which further confirmed our hypothesis. Meanwhile, the correlation analysis showed that Qi-deficiency score, Blood-stasis score, and Qi-depression score were also positively correlated with SAS, SDS, and PSQI scores. Interestingly, the Qi-depression score had the relatively best predictive ability for migraine, with an AUC of 0.76 (95% CI: 0.68–0.84). According to the correlation cluster graph (Fig. 5), the Qi-depression score was also demonstrated as a most correlated TCMC score with SAS score (*r* = 0.56) and SDS score (*r* = 0.60), which might indirectly explain the close relationship between Qi-depression score with the risk of developing migraine. From the concept of TCM, people with Qi-depression constitution usually behave depression, nervousness, fear or sigh for no reason, which is very similar to the anxiety or depression co-morbidities of migraine [7]. People with Qi-deficiency constitution are mainly characterized by lethargy, tiredness, and are more common in women, and this is very similar to the sleepiness and fatigue in the triggers of migraine attack [51]. People with blood stasis are often characterized by relative stagnation of blood in the local area of the body, resulting in pain, and this relative stagnation of blood stasis is often caused by abnormal blood flow or abnormal vasoconstriction and diastole, which is also similar to the pathogenesis of migraine [4].

To develop the ideal prediction models for migraine, this study preliminarily developed model 1 and model 2, including one indicator of PSQI score and two indicators of PSQI score and Qi-depression score, with the AUC of 0.83 (95% CI: 0.77–0.89) and 0.86 (95% CI: 0.80—0.91), respectively. On this basis, we further developed models 3 to 8 including 3 to 8 indicators, and the results showed that the predictive performance of these models for migraine were further improved, with AUCs ranging from 0.87 (95% CI: 0.82–0.92) to 0.92 (95% CI: 0.89–0.96). Compared with the single best predictor, PSQI score, the predictive accuracy from Model 1 to Model 8 increased by 51.1%, 47.6%, 54.0%, 52.4%, 64.0%, 59.1%, 61.5%, and 72.2%, respectively, which suggested that the models developed in this study were significantly better than PSQI score for migraine. Moreover, 7 of these 8 models included TCMC scores, which also indicated indirectly that the inclusion of TCMC scores improved the overall performance of the models. It was worth noting that Model 6 had the best specificity (0.95) indicating the lowest missed diagnosis rate, while Model 8 had the best sensitivity (0.92) indicating the lowest misdiagnosis rate. In clinical practice, different prediction models could be selected based on different application scenarios.

In terms of the calibration of the models, the study showed that Model 8, which included eight variables, was a weaker calibration curve than the other models, which may have been caused by the addition of the variables resulting in a relatively small sample size for the statistical analysis of the logistic regression. But despite this, Model 8 remained a strong predictor of migraine in the validation set, with an AUC of 0.82 (95% CI: 0.74–0.90). Meanwhile, Models 1 through 7 were both well discriminated and well calibrated and also demonstrated good predictive ability for migraine in the validation set, with AUCs ranging from 0.76 (95% CI: 0.66–0.85) to 0.83 (95% CI: 0.75–0.91). In addition, the predict outcomes of eight models in both the training and validation sets were significantly and independently positively associated with the actual occurrence of migraine, which further confirmed the excellent predictive performance of the eight prediction models for migraine.

Considering the epidemiological differences of migraine in terms of gender and age [3, 4], the predictive ability of various models for migraine of different genders and ages were worth further exploration. From the ROC curves, it was known that in the training set, there was no significant difference in the predictive ability of the models for migraine in men and women, while in the validation set there was a tendency for the models to have a higher predictive ability for men than for women. The reason for this may be due to the relatively small sample size of male subjects in the validation set, which may amplify the positive results of gender differences. Nonetheless, we did not believe that the models were not applicable to males but only to females. Because the results of the subgroup analyses showed that the AUCs ranged from 0.80 (95% CI: 0.63—0.97) to 0.95 (95% CI: 0.91–1.00) for the different ages and genders in the training set, and ranged from 0.73 (95% CI: 0.64–0.84) to 0.93 (95% CI: 0.82–1.00) in the validation set, with all AUCs greater than 0.7, further validating that these models were applicable to migraine patients of different ages and genders with good predictive performance.

Compared to previous studies with invasive or high-cost biomarker studies for predicting migraine, the noninvasive predictive models in this study has the advantages of being easier to access and less costly. In addition, compared with other clinical subjective descriptive diagnostic models, this study used specialized scales to more objectively assess the PSQI, SAS, and SDS scores that are closely related to the predictors of migraine, and combined them with the TCMC scores to establish novel prediction models, which makes the prediction results more accurate and more suitable for efficiently predicting and screening the population of potential migraine risk. It is particularly suitable for early warning of migraine risk for community hospitals and homes with relatively inadequate medical care, or for people at high risk of migraine with sleep and mood disorders. In the future, we hope to develop our noninvasive prediction model into a portable mobile application for early screening and timely advice and intervention for migraine in Chinese adults.

In summary, this study developed a series of convenient and novel non-invasive prediction models for migraine, and conducted external validation and subgroup analysis, confirming that the excellent predictive ability of these prediction models for migraine in Chinese adults of different genders and ages. It was of great significance for early prevention, screening, and diagnosis of migraine. However, this study also had some limitations. Firstly, the participants in the training and validation sets of this study were from the single clinical center, although the enrollment time was different, there might still be a selection bias. In the future, further external validation needs to be conducted by including participants in different regions and clinical application scenarios. Secondly, when exploring subgroup analysis, the sample size of male subjects in this study was relatively small, although the minimum sample size required for statistics was reached, positive results of gender differences may be amplified due to selection bias. Therefore, we hope to further expand the sample size in the future to validate the gender difference of prediction models for migraine. Thirdly, the main research indicators of this study were obtained through questionnaire scales, although they were all filled out under the guidance of professional doctors, there may still be some subjectivity. In the future, we hope to incorporate more objective laboratory biochemical markers, imaging markers, etc., and construct a series of more diversified migraine prediction models to achieve more precise prediction for migraine.

## Conclusions

This study developed and validated a series of convenient and novel non-invasive prediction models for migraine, which have good predictive ability for migraine in Chinese adults of different genders and ages. It is of great significance for the early prevention, screening, diagnosis of migraine.

### Supplementary Information

**Additional file1:** **Supplementary Table 1.** NRI analysis between Model 1 and PSQI score predictor in the training set. **Supplementary Table 2.** NRI analysis between Model 2 and PSQI score predictor in the training set. **Supplementary Table 3.** NRI analysis between Model 3 and PSQI score predictor in the training set. **Supplementary Table 4.** NRI analysis between Model 4 and PSQI score predictor in the training set. **Supplementary Table 5.** NRI analysis between Model 5 and PSQI score predictor in the training set. **Supplementary Table 6.** NRI analysis between Model 6 and PSQI score predictor in the training set. **Supplementary Table 7.** NRI analysis between Model 7 and PSQI score predictor in the training set. **Supplementary Table 8.** NRI analysis between Model 8 and PSQI score predictor in the training set. **Supplementary Table 9.** NRI analysis between Model 1 and PSQI score predictor in the validation set. **Supplementary Table 10.** NRI analysis between Model 2 and PSQI score predictor in the validation set. **Supplementary Table 11.** NRI analysis between Model 3 and PSQI score predictor in the validation set. **Supplementary Table 12.** NRI analysis between Model 4 and PSQI score predictor in the validation set.** Supplementary Table 13.** NRI analysis between Model 5 and PSQI score predictor in the validation set. **Supplementary Table 14.** NRI analysis between Model 6 and PSQI score predictor in the validation set. **Supplementary Table 15.** NRI analysis between Model 7 and PSQI score predictor in the validation set. **Supplementary Table 16.** NRI analysis between Model 8 and PSQI score predictor in the validation set.

**Additional file 2.** Reporting checklist for prediction model development/validation.

## Data Availability

The datasets used and analyzed during the present study are available from the corresponding authors on reasonable request.

## Abbreviations

SAS: Self-rating anxiety scale
SDS: Self-rating depression scale
PSQI: Pittsburgh sleep quality index
TCMC: Traditional Chinese medicine constitution
ROC: Receiver operating characteristic
AUC: Area under the ROC curve
ICHD-III: International classification of headache disorders, 3rd edition
BMI: Body mass index
VAS: Visual analog scale
HIT-6: Headache impact test
MIDAS: Migraine disability assessment
MSQ: Migraine-specific quality-of-life questionnaire
SD: Standard deviation
IQR: Interquartile range
