# Supplementary material for: Development and validation of non-invasive prediction models for migraine in Chinese adults
Source: J Headache Pain. 2023 Nov 6;24(1):148. doi: 10.1186/s10194-023-01675-1 (PMC10626650; doi:10.1186/s10194-023-01675-1)
Supplement: Supplementary file 2 — Additional file 2. Reporting checklist for prediction model development/validation. [file 10194_2023_1675_MOESM2_ESM.docx]

**Supplementary Tables**

**Supplementary table 1 NRI analysis between Model 1 and PSQI score predictor in the training set.**

| Model 1 | Estimate | Std.Error | Lower | Upper |
| --- | --- | --- | --- | --- |
| NRI | **0.511** | 0.07 | 0.373 | 0.643 |
| NRI+ | -0.172 | 0.033 | -0.238 | -0.109 |
| NRI- | 0.683 | 0.061 | 0.562 | 0.8 |
| Pr(Up\|Case) | 0 | 0 | 0 | 0 |
| Pr(Down\|Case) | 0.172 | 0.033 | 0.109 | 0.238 |
| Pr(Down\|Ctrl) | 0.683 | 0.061 | 0.562 | 0.8 |
| Pr(Up\|Ctrl) | 0 | 0 | 0 | 0 |

**Supplementary table 2 NRI analysis between Model 2 and PSQI score predictor in the training set.**

| Model 2 | Estimate | Std.Error | Lower | Upper |
| --- | --- | --- | --- | --- |
| NRI | **0.476** | 0.07 | 0.337 | 0.612 |
| NRI+ | -0.141 | 0.031 | -0.205 | -0.083 |
| NRI- | 0.617 | 0.063 | 0.491 | 0.738 |
| Pr(Up\|Case) | 0 | 0 | 0 | 0 |
| Pr(Down\|Case) | 0.141 | 0.031 | 0.083 | 0.205 |
| Pr(Down\|Ctrl) | 0.617 | 0.063 | 0.491 | 0.738 |
| Pr(Up\|Ctrl) | 0 | 0 | 0 | 0 |

**Supplementary table 3 NRI analysis between Model 3 and PSQI score predictor in the training set.**

| Model 3 | Estimate | Std.Error | Lower | Upper |
| --- | --- | --- | --- | --- |
| NRI | **0.540** | 0.067 | 0.404 | 0.668 |
| NRI+ | -0.094 | 0.026 | -0.148 | -0.047 |
| NRI- | 0.633 | 0.062 | 0.507 | 0.754 |
| Pr(Up\|Case) | 0 | 0 | 0 | 0 |
| Pr(Down\|Case) | 0.094 | 0.026 | 0.047 | 0.148 |
| Pr(Down\|Ctrl) | 0.633 | 0.062 | 0.507 | 0.754 |
| Pr(Up\|Ctrl) | 0 | 0 | 0 | 0 |

**Supplementary table 4 NRI analysis between Model 4 and PSQI score predictor in the training set.**

| Model 4 | Estimate | Std.Error | Lower | Upper |
| --- | --- | --- | --- | --- |
| NRI | **0.524** | 0.068 | 0.391 | 0.658 |
| NRI+ | -0.109 | 0.028 | -0.167 | -0.059 |
| NRI- | 0.633 | 0.063 | 0.509 | 0.758 |
| Pr(Up\|Case) | 0 | 0 | 0 | 0 |
| Pr(Down\|Case) | 0.109 | 0.028 | 0.059 | 0.167 |
| Pr(Down\|Ctrl) | 0.633 | 0.063 | 0.509 | 0.758 |
| Pr(Up\|Ctrl) | 0 | 0 | 0 | 0 |

**Supplementary table 5 NRI analysis between Model 5 and PSQI score predictor in the training set.**

| Model 5 | Estimate | Std.Error | Lower | Upper |
| --- | --- | --- | --- | --- |
| NRI | **0.640** | 0.062 | 0.515 | 0.759 |
| NRI+ | -0.094 | 0.026 | -0.147 | -0.047 |
| NRI- | 0.733 | 0.057 | 0.619 | 0.841 |
| Pr(Up\|Case) | 0 | 0 | 0 | 0 |
| Pr(Down\|Case) | 0.094 | 0.026 | 0.047 | 0.147 |
| Pr(Down\|Ctrl) | 0.733 | 0.057 | 0.619 | 0.841 |
| Pr(Up\|Ctrl) | 0 | 0 | 0 | 0 |

**Supplementary table 6 NRI analysis between Model 6 and PSQI score predictor in the training set.**

| Model 6 | Estimate | Std.Error | Lower | Upper |
| --- | --- | --- | --- | --- |
| NRI | **0.591** | 0.066 | 0.459 | 0.715 |
| NRI+ | -0.109 | 0.028 | -0.167 | -0.058 |
| NRI- | 0.7 | 0.06 | 0.582 | 0.812 |
| Pr(Up\|Case) | 0 | 0 | 0 | 0 |
| Pr(Down\|Case) | 0.109 | 0.028 | 0.058 | 0.167 |
| Pr(Down\|Ctrl) | 0.7 | 0.06 | 0.582 | 0.812 |
| Pr(Up\|Ctrl) | 0 | 0 | 0 | 0 |

**Supplementary table 7 NRI analysis between Model 7 and PSQI score predictor in the training set.**

| Model 7 | Estimate | Std.Error | Lower | Upper |
| --- | --- | --- | --- | --- |
| NRI | **0.615** | 0.064 | 0.488 | 0.735 |
| NRI+ | -0.102 | 0.027 | -0.157 | -0.053 |
| NRI- | 0.717 | 0.058 | 0.6 | 0.825 |
| Pr(Up\|Case) | 0 | 0 | 0 | 0 |
| Pr(Down\|Case) | 0.102 | 0.027 | 0.053 | 0.157 |
| Pr(Down\|Ctrl) | 0.717 | 0.058 | 0.6 | 0.825 |
| Pr(Up\|Ctrl) | 0 | 0 | 0 | 0 |

**Supplementary table 8 NRI analysis between Model 8 and PSQI score predictor in the training set.**

| Model 8 | Estimate | Std.Error | Lower | Upper |
| --- | --- | --- | --- | --- |
| NRI | **0.722** | 0.058 | 0.604 | 0.83 |
| NRI+ | -0.078 | 0.023 | -0.126 | -0.036 |
| NRI- | 0.8 | 0.053 | 0.691 | 0.898 |
| Pr(Up\|Case) | 0 | 0 | 0 | 0 |
| Pr(Down\|Case) | 0.078 | 0.023 | 0.036 | 0.126 |
| Pr(Down\|Ctrl) | 0.8 | 0.053 | 0.691 | 0.898 |
| Pr(Up\|Ctrl) | 0 | 0 | 0 | 0 |

**Supplementary table 9 NRI analysis between Model 1 and PSQI score predictor in the validation set.**

| Model 1 | Estimate | Std.Error | Lower | Upper |
| --- | --- | --- | --- | --- |
| NRI | **0.425** | 0.096 | 0.234 | 0.605 |
| NRI+ | -0.375 | 0.06 | -0.492 | -0.258 |
| NRI- | 0.8 | 0.074 | 0.647 | 0.933 |
| Pr(Up\|Case) | 0 | 0 | 0 | 0 |
| Pr(Down\|Case) | 0.375 | 0.06 | 0.258 | 0.492 |
| Pr(Down\|Ctrl) | 0.8 | 0.074 | 0.647 | 0.933 |
| Pr(Up\|Ctrl) | 0 | 0 | 0 | 0 |

**Supplementary table 10 NRI analysis between Model 2 and PSQI score predictor in the validation set.**

| Model 2 | Estimate | Std.Error | Lower | Upper |
| --- | --- | --- | --- | --- |
| NRI | **0.474** | 0.091 | 0.289 | 0.646 |
| NRI+ | -0.359 | 0.061 | -0.483 | -0.243 |
| NRI- | 0.833 | 0.068 | 0.69 | 0.962 |
| Pr(Up\|Case) | 0 | 0 | 0 | 0 |
| Pr(Down\|Case) | 0.359 | 0.061 | 0.243 | 0.483 |
| Pr(Down\|Ctrl) | 0.833 | 0.068 | 0.69 | 0.962 |
| Pr(Up\|Ctrl) | 0 | 0 | 0 | 0 |

**Supplementary table 11 NRI analysis between Model 3 and PSQI score predictor in the validation set.**

| Model 3 | Estimate | Std.Error | Lower | Upper |
| --- | --- | --- | --- | --- |
| NRI | **0.421** | 0.1 | 0.217 | 0.609 |
| NRI+ | -0.312 | 0.059 | -0.433 | -0.203 |
| NRI- | 0.733 | 0.081 | 0.567 | 0.883 |
| Pr(Up\|Case) | 0 | 0 | 0 | 0 |
| Pr(Down\|Case) | 0.312 | 0.059 | 0.203 | 0.433 |
| Pr(Down\|Ctrl) | 0.733 | 0.081 | 0.567 | 0.883 |
| Pr(Up\|Ctrl) | 0 | 0 | 0 | 0 |

**Supplementary table 12 NRI analysis between Model 4 and PSQI score predictor in the validation set.**

| Model 4 | Estimate | Std.Error | Lower | Upper |
| --- | --- | --- | --- | --- |
| NRI | **0.536** | 0.09 | 0.352 | 0.702 |
| NRI+ | -0.297 | 0.057 | -0.412 | -0.188 |
| NRI- | 0.833 | 0.069 | 0.688 | 0.962 |
| Pr(Up\|Case) | 0 | 0 | 0 | 0 |
| Pr(Down\|Case) | 0.297 | 0.057 | 0.188 | 0.412 |
| Pr(Down\|Ctrl) | 0.833 | 0.069 | 0.688 | 0.962 |
| Pr(Up\|Ctrl) | 0 | 0 | 0 | 0 |

**Supplementary table 13 NRI analysis between Model 5 and PSQI score predictor in the validation set.**

| Model 5 | Estimate | Std.Error | Lower | Upper |
| --- | --- | --- | --- | --- |
| NRI | **0.485** | 0.096 | 0.29 | 0.665 |
| NRI+ | -0.281 | 0.057 | -0.397 | -0.175 |
| NRI- | 0.767 | 0.077 | 0.607 | 0.909 |
| Pr(Up\|Case) | 0 | 0 | 0 | 0 |
| Pr(Down\|Case) | 0.281 | 0.057 | 0.175 | 0.397 |
| Pr(Down\|Ctrl) | 0.767 | 0.077 | 0.607 | 0.909 |
| Pr(Up\|Ctrl) | 0 | 0 | 0 | 0 |

**Supplementary table 14 NRI analysis between Model 6 and PSQI score predictor in the validation set.**

| Model 6 | Estimate | Std.Error | Lower | Upper |
| --- | --- | --- | --- | --- |
| NRI | **0.452** | 0.099 | 0.249 | 0.639 |
| NRI+ | -0.281 | 0.057 | -0.397 | -0.174 |
| NRI- | 0.733 | 0.082 | 0.567 | 0.886 |
| Pr(Up\|Case) | 0 | 0 | 0 | 0 |
| Pr(Down\|Case) | 0.281 | 0.057 | 0.174 | 0.397 |
| Pr(Down\|Ctrl) | 0.733 | 0.082 | 0.567 | 0.886 |
| Pr(Up\|Ctrl) | 0 | 0 | 0 | 0 |

**Supplementary table 15 NRI analysis between Model 7 and PSQI score predictor in the validation set.**

| Model 7 | Estimate | Std.Error | Lower | Upper |
| --- | --- | --- | --- | --- |
| NRI | **0.452** | 0.1 | 0.251 | 0.644 |
| NRI+ | -0.281 | 0.056 | -0.397 | -0.173 |
| NRI- | 0.733 | 0.082 | 0.565 | 0.882 |
| Pr(Up\|Case) | 0 | 0 | 0 | 0 |
| Pr(Down\|Case) | 0.281 | 0.056 | 0.173 | 0.397 |
| Pr(Down\|Ctrl) | 0.733 | 0.082 | 0.565 | 0.882 |
| Pr(Up\|Ctrl) | 0 | 0 | 0 | 0 |

**Supplementary table 16 NRI analysis between Model 8 and PSQI score predictor in the validation set.**

| Model 8 | Estimate | Std.Error | Lower | Upper |
| --- | --- | --- | --- | --- |
| NRI | **0.483** | 0.098 | 0.28 | 0.668 |
| NRI+ | -0.25 | 0.055 | -0.361 | -0.143 |
| NRI- | 0.733 | 0.082 | 0.565 | 0.889 |
| Pr(Up\|Case) | 0 | 0 | 0 | 0 |
| Pr(Down\|Case) | 0.25 | 0.055 | 0.143 | 0.361 |
| Pr(Down\|Ctrl) | 0.733 | 0.082 | 0.565 | 0.889 |
| Pr(Up\|Ctrl) | 0 | 0 | 0 | 0 |
